# Editorial: Long-term well-being and mental health in a public health emergency

**DOI:** 10.3389/fpsyt.2024.1433053

**Published:** 2024-06-06

**Authors:** Grace Yao Jie Xie, Gerard A. Kennedy, Grace W. K. Ho, Francisco Sampaio

**Affiliations:** ^1^ School of Nursing, The Hong Kong Polytechnic University, Hong Kong, Hong Kong SAR, China; ^2^ Institute for Health and Wellbeing, Psychology, Federation University, Ballarat, VIC, Australia; ^3^ Nursing School of Porto, Porto, Portugal; ^4^ CINTESIS@RISE, Nursing School of Porto (ESEP), Porto, Portugal

**Keywords:** mental health, public health, emergencies, COVID-19, pandemics, resilience

The COVID-19 pandemic has led to significant global changes in public health priorities, with mental health issues now ranking among the top concerns alongside more conventional medical problems (1). The impacts of the pandemic were universally felt and disrupted well-being across individual, community, and systems levels. On an unprecedented scale, people across the globe participated in some level of social distancing and isolation and endured the fear and uncertainty of an emerging disease. Communities were challenged to respond to an ever-changing environment within the confines of the resources and technologies available to them and, in many cases, demonstrated agility and resilience. Health care systems, in particular, faced significant problems in the face of a pandemic of this magnitude. It put vulnerable populations and healthcare staff at serious risk, and hospitals were pushed to the edge of their ability to care for patients (2). The COVID-19 pandemic was a collective human experience that has affected the mental well-being of all.

To shed light on the complex and largely uncharted environment of the COVID-19 pandemic, as well as other public health emergencies (e.g., Monkeypox, Ebola, earthquakes, tsunamis), the Research Topic “Long-term Well-being and Mental Health in a Public Health Emergency” has assembled a thoughtful selection of papers that explore the long-term effects of such events on psychological health to help us understand how the social and environmental determinants contribute to the progression of the individual’s psychological health. Although these investigations were conducted against the backdrop of the COVID-19 pandemic, the findings are expected to inform the preparation and considerations in future public health emergencies so as to safeguard the long-term mental well-being of those affected.

The collection of articles show-cased a wide range of research initiatives, each offering a distinct perspective that advances our comprehension of the relationship between mental health and public health issues. Luo et al., for example, provided a thorough examination of the psychological effects of the COVID-19 quarantine, revealing the complex relationship between social isolation and negative mental health consequences. Meanwhile, Morris et al. investigated how the pandemic has affected vulnerable communities differently, highlighting the necessity of focused measures to reduce inequalities in mental health service delivery and improve outcomes.

The concept of resilience in the face of adversity is mentioned frequently in these studies. In order to manage times of increased stress, people can use their personal strengths, as shown by Chen et al., who provided important insights into the protective variables like resilience that can support mental health during emergencies. These findings have significant ramifications for public health policy and intervention strategies in addition to being rigorously empirical.

The psychological consequences of the pandemic on healthcare workers have also been studied by our contributors. Rachubińska et al., for instance, revealed how the COVID-19 pandemic affected nurses’ psychological health, especially in terms of elevated stress and anxiety symptoms. Wang et al. reported that mindfulness-based training appears to be a promising strategy for enhancing nurses’ psychological health, including lowering stress and reducing burnout. Liu et al. found that nurses showed moderate levels of knowledge, relatively positive attitudes, and good practice regarding occupational exposure in public health emergencies. These results are consistent with several other studies conducted during the COVID-19 pandemic that showed greater levels of stress, anxiety, and depression symptoms among nurses, despite the observation of a psychological adaptation phenomenon (3, 4).

Studies have also been conducted on some other specific populations, including youth (students in grades 7–12) and prison guards. Despite the COVID-19 pandemic lockdowns, students largely demonstrated stable mental health prior to, during, and following their return to in-person classroom instruction, as shown by the findings of research conducted by Qian et al. However, a study by Zeng et al. found that, during the protracted COVID-19 pandemic, anxiety and depression were relatively common among Chinese prison officers, especially for those with work-family conflicts, high job demands, and COVID-19 burnout. This study offers the prison system a guide to enhance prison officers’ mental health during future closed-loop management periods.

This compilation of research papers makes it abundantly evident that the conversation on public health emergencies needs to go beyond the scope of just a medical emergency to include both mental health and community well-being in its entirety (5). Leveraging interdisciplinary insights and forming synergistic collaborations that bridge the gap between clinical practice and mental health advocacy becomes crucial as we negotiate the complexity of this rapidly evolving area. If this is done, then there can be better preparedness if and when we are faced with another pandemic event.

The collection of papers in this Research Topic serves as a more than timely wake-up call for the funding of long-term mental health programs (6), which are primarily targeted at particular demographic groups (First Nations people, cultural minorities, people of lower socioeconomic status, older people, people with preexisting mental conditions, and sexual and gender minority groups) who seemed to be particularly vulnerable during the COVID-19 pandemic (7). Through utilizing the full breath of combined knowledge contained in these articles, we may influence public health agendas and policy in the direction of a more comprehensive and inclusive model that prioritizes mental health.

To sum up, the articles featured in this Research Topic highlight the significant negative effects events such as the COVID-19 pandemic can have on mental health, making them a fundamental contribution to and a useful resource for the developing field of public mental health and disaster preparedness specifically and mental health promotion and disease prevention on a larger scale. Importantly, the consensus was that, with knowledge, preparation, and support, humans can be incredibly resilient in the face of new and emerging health threats. Let us use these academic efforts as motivation to work towards a society in which psychological resilience is a concrete reality rather than just an ideal as we face the problems of the future.

In the face of the demands of our days, we encourage readers to peruse this research topic and to stand with us in our endeavor to foster a generous, compassionate, and understanding society.

## Author contributions

GX: Writing – review & editing. GK: Writing – review & editing. GH: Writing – review & editing. FS: Conceptualization, Writing – original draft, Writing – review & editing.
